# MRI characteristics of proctitis in Crohn’s disease on perianal MRI

**DOI:** 10.1007/s00261-016-0802-z

**Published:** 2016-06-17

**Authors:** Charlotte J. Tutein Nolthenius, Shandra Bipat, Banafsche Mearadji, Anje M. Spijkerboer, Cyriel Y. Ponsioen, Alexander D. Montauban van Swijndregt, Jaap Stoker

**Affiliations:** 1 Department of Radiology, Academic Medical Center, University of Amsterdam, PO Box 22660, 1100 DD Amsterdam, The Netherlands; 2 Department of Gastroenterology and Hepatology, Academic Medical Center, University of Amsterdam, PO Box 22700, 1100 DE Amsterdam, The Netherlands; 3 Department of Radiology, OLVG, PO Box 99550, 1090 HM Amsterdam, The Netherlands

**Keywords:** Proctitis, Crohn disease, Magnetic resonance imaging, Rectum, Inflammatory bowel disease

## Abstract

**Purpose:**

Multiple features have been described for assessing inflammation in Crohn’s disease (CD) in MR enterography, but have not been validated in perianal magnetic resonance imaging (MRI). Retrospectively, we studied which MRI features are valuable in assessing proctitis.

**Materials and methods:**

CD patients (≥18 years) who underwent colonoscopy (reference standard) and perianal fistula MRI within 8 weeks were included. Seventeen MRI features were blindly scored by three observers and correlated to endoscopy (regression analysis). Reproducibility (multirater kappa, intraclass correlation coefficient) was determined for all three observer pairs. MRI features were considered relevant when significantly correlated to endoscopy for ≥2 observers, and reproducibility was ≥0.40 for ≥2 observer pairs.

**Results:**

Perianal MRI of 58 CD patients were included. Wall thickness, rectal mural fat, creeping fat, and size of mesorectal lymph nodes showed a significant correlation with endoscopy for ≥2 observers (*p* = 0.000–0.023, *p* = 0.011–0.172, *p* = 0.007–0.011 and *p* = 0.000–0.005, respectively) with a kappa/intraclass correlation coefficient of ≥0.60 for ≥2 observer pairs. Perimural T2 signal and perimural enhancement significantly correlated to endoscopy (all *p* values ≤0.05) for all three observers and the reproducibility was ≥0.40 for ≥2 observer pairs. Mural T2 signal and degree and pattern of T1 enhancement showed significant correlation to endoscopy for two observers, but with poor to moderate reproducibility.

**Conclusion:**

Wall thickness, mural fat, and mesorectal features (perimural T2 signal, perimural enhancement, creeping fat, and size of mesorectal lymph nodes) had significant correlation to endoscopy and were reproducible in diagnosing proctitis. Some established luminal features in MRE were considered not useful.

Magnetic resonance imaging (MRI) of the perianal region has proven to be a valuable tool in diagnosing perianal fistulas in patients with Crohn’s disease, with accuracies reported up to 93% in classifying fistulas and 96% in delineating abscesses [1, 2]. The anatomy and complexity of the fistula tract can precisely be depicted which is important for treatment planning [3]. Preoperative MRI has shown to reveal additional and clinically relevant information, thereby reducing recurrence rates after fistula surgery [1, 4]. Another important issue in treatment planning is the concomitant presence of proctitis. Proctitis is defined as an inflammation of the rectum, approximately 12–15 cm from the dentate line. In the presence of proctitis, the chance of fistula healing is reduced, and therefore, a more aggressive medical therapy should be started and surgery should be avoided [3, 5, 6].

 Extensive research revealed multiple MRI features and scoring systems able to accurately assess inflammation in luminal Crohn’s disease [7–9]. These features have been assessed on MR enterography or MR colonography, and have not been tested in dedicated pelvic MRI, which is limited by a different scan protocol (small FOV, other sequences) and the absence of luminal contrast. As many patients with perianal fistulas will undergo a pelvic MRI before start of treatment, diagnosing the presence and degree of proctitis on this MRI could be of additional value [2, 3].

In our retrospective study, we aimed to identify the MRI features of proctitis on a dedicated pelvic MRI, and to determine the reproducibility of the different MRI features.

## Materials and methods

### Patients

From January 2001 until February 2014, we searched the endoscopy database (EndoAlpha Documentation, Olympus Nederland BV, Zoeterwoude, The Netherlands) of the Academic Medical Center, Amsterdam, The Netherlands, for patients (≥18 years of age) with known Crohn’s disease who underwent a proctoscopy, sigmoidoscopy, or colonoscopy and who also underwent a dedicated pelvic MRI according to our standard MRI perianal fistula protocol within either 8 weeks prior to or after endoscopy. We chose this eight-week interval balancing inclusion versus a satisfactory interval. Patients were included if the endoscopy report mentioned the rectum, either with regard to the diagnosis of proctitis, rectal inflammation, or rectitis or with regard to no signs of rectal inflammation at all. Patients could only be included once. In that case, the most recent MRI was chosen. For consistency, MRIs performed with an endocoil or with an incomplete scan protocol were excluded. Electronic medical records were searched by a research fellow (CTN) and relevant information was noted (time of diagnosis, use of medication during the examinations, previous surgery). Patients with change in therapy, either medical or surgical, in the period between endoscopy and MRI were excluded. All included MRI scans were blinded and randomly ordered.

The requirement for review by the Medical Ethical Committee or informed consent was waived because of the retrospective nature of this study with pre-existing data.

### Reference standard

With no access to clinical information or MRI scans, we evaluated the endoscopy reports of all included patients and performed a classification of lesion severity by considering three categories: grade (1) absence of lesions; grade (2) presence of inflammatory lesions without ulceration, including erythema, oedema, pseudopolyps, and aphthae; and grade (3) presence of superficial or deep ulcerations [10]. The presence or absence of fistulas and anal stenosis was also noted. Uncertainties were resolved by the expert opinion of a gastroenterologist (CY; 20 years of experience), with inflammatory bowel disease as subspecialty, with access to all endoscopical information, including endoscopy images.

### MRI protocol

All MRIs were performed at 1.5T (Signa Horizon Echospeed, LX 9.0, General Electric Medical Systems, Milwaukee, WI, USA and MAGNETOM Avanto, Siemens Healthcare, Erlangen, Germany) and at 3T (INTERA, Philips Medical Systems, Best, the Netherlands) without bowel preparation, except 4-h fasting. Patients were scanned in supine position using a torso phased-array surface coil. Sagittal, coronal, and transversal sequences were performed with the coronal and transversal sequences angulated parallel and perpendicular to the anal canal, respectively. The scan protocol consisted of T2-weighted Turbo Spin-Echo sequences in the sagittal, coronal, and transversal planes, a fat-suppressed transversal T2-weighted TSE sequence and a fat-suppressed transversal T1-weighted TSE sequence after intravenous gadolinium. For a detailed description of all MRI parameters see Appendix A.

### Observers

**Fig. 1:**
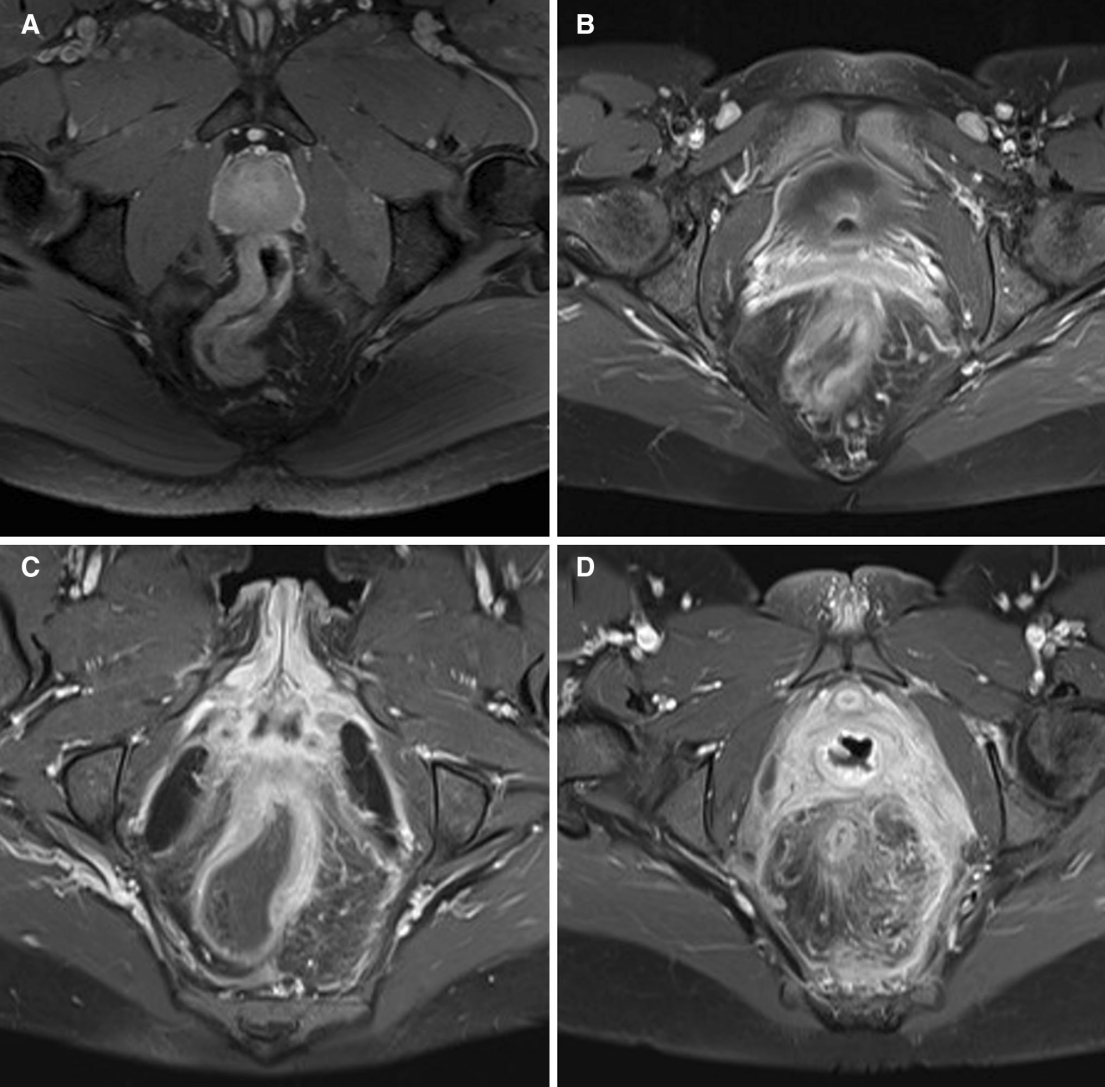
Axial oblique fat-saturated post-contrast T1-weighted images of four different patients with Crohn’s disease with different degrees of perimural enhancement. **A** Equivalent to normal fat tissue. **B** Minor enhancement. There is blurred demarcation of the bowel wall with or without mild increase of perimural fat tissue signal. **C** Moderate enhancement. Increase of perimural fat tissue signal but less than nearby vascular structures. **D** Marked enhancement. Perimural fat tissue signal approaches that of nearby vascular structures. Mesorectal fascia enhancement can be noted

All MRI scans were blinded and retrospectively evaluated by three abdominal radiologists with different relevant experience levels: Observer 1 (BM; abdominal radiologist for 9 years including approximately 500 perianal fistula MRIs and 500 MR enterographies) years, observer 2 (AS; radiologist for 20 years including approximately 300 perianal fistula MRIs and 300 MR enterographies), and observer 3 (JS; abdominal radiologist for 21 years including approximately 1300 perianal fistula MRIs and 800 MR enterographies). No clinical or endoscopy findings were provided, except for the presence or absence of perianal fistulas. . Before start, the observers read a document explaining the different MRI features to be evaluated with examples obtained from the previous literature and cases from a different dataset followed by a joined session discussing the features led by a fellow researcher (CTN) and the most experienced abdominal radiologist (observer 3: JS) (Fig. 1) [7, 11–14]. Five example cases from a different dataset were discussed. For evaluation of all cases, a digital questionnaire was developed (proctitis.co.nr).

### MRI features

Quality of the scan and rectal distention were evaluated by the most experienced observer as poor (non-diagnostic), adequate (artifacts, but sufficient diagnostic quality), and good (no artifacts); and none (completely collapsed rectum), moderate (some distension but no convex contours of the rectal wall), and good (convex contours of the rectal wall) assessed in the least distended part of the rectum, respectively. Seventeen MRI features (Table 1) were evaluated by all three readers. Features were selected according to MRI features described in the literature and those used in two published scoring systems on MRI in luminal Crohn’s disease [7, 10, 11]. Definitions of certain features were adapted to be applicable in perianal MRI (Table 1). Additional items according to expert opinion were added: enhancement of perimural fat tissue (see Table 1; Fig. 1 for definition), creeping fat was defined as an increased amount of perirectal fat tissue and the comb sign as increased vascular structures in the perirectal fat, both scored on the sagittal images. The most affected part of the rectum was evaluated. Shortest axis of the largest lymph node per station was measured.

**Table 1:** MRI features, evaluated in the most affected part of the rectum

Wall thickness in mm^a^				
Largest regional lymph node in mm (mesorectal, obturator, iliac, inguinal)				
% of circumference involved	0–25%	26–50%	51–75%	76–100%
Mural T2 signal^b^	Equivalent to normal bowel wall	Minor increase—bowel wall appears dark gray on fat-saturated images	Moderate increase—bowel wall appears light gray on fat-saturated images	Marked increase—bowel wall contains areas of white high signal approaching that of nearby vascular structures
Perimural T2 signal^b^	Equivalent to normal fat tissue	Increase in signal but no fluid	Small fluid rim (≤2 mm)	Larger fluid rim (>2 mm)
T1 enhancement^b^	Equivalent to normal bowel wall	Minor enhancement—bowel wall signal increased but significantly less than nearby vascular structures	Moderate enhancement—bowel wall signal increased but somewhat less than nearby vascular structures	Marked enhancement—bowel wall signal approaches that of nearby vascular structures
T1 enhancement pattern^b^	N/A^c^	Homogeneous	Mucosal	Layered
Enhancement of perimural fat tissue	Equivalent to normal fat tissue	Minor enhancement—blurred demarcation of the bowel wall with/without mild increase of perimural fat tissue signal	Moderate enhancement—increase of perimural fat tissue signal but less than nearby vascular structures	Marked enhancement—perimural fat tissue signal approaches that of nearby vascular structures. Mesorectal fascia enhancement can be noted
Mural fat	Absent	Present		
Ulcers^a^	Absent	Present		
Supralevatoric fistula	Absent	Present		
Supralevatoric abscess	Absent	Present		
Creeping fat	Absent	Present		
Comb sign	Absent	Present		

^a^According to Rimola et al. IBD 2011

^b^According to Steward et al EJR 2012

^c^ *N/A* in case of enhancement equivalent to normal bowel wall

### Statistical analysis

The maximum number of eligible patients in the given time period were included, and no sample size calculations were therefore performed. Extension of this time period to earlier period was not desirable, as the MRI protocol was different before 2001 (use of endocoil) and thereby not reflecting the practice nowadays.

Normality of continuous data was tested by visual assessment of the data. Normally distributed data were presented with means and SD. For non-normally distributed data, medians with interquartile ranges (IQR) were given.

### Interobserver agreement

Several multirater analyses were performed for all features individually. To test the level of interobserver agreement for the separate MRI features between the three different pairs of radiologists, the appropriate measure was used. For all ordinal data, a weighted kappa coefficient was calculated per two raters. For the binominal data, a kappa coefficient was used calculated per two raters. For continuous data, an intraclass correlation coefficient was determined per two raters. Both kappa and intraclass correlation coefficient values were interpreted as follows: 0–0.20, poor; 0.20–0.40, fair; 0.40–0.60, moderate; 0.60–0.80, good; 0.80–1.00, very good [15].

### Comparison of observers with reference standard

Endoscopical reference standard was dichotomized in the absence of lesions (grade 0) versus proctitis (grades 1 and 2) because of limited size of study population. Associations were tested using regression analyses for ordinal or binominal MRI parameters. Comparison of continuous MRI parameters and endoscopical reference standard was performed using the Mann–Whitney *U* test, as data were not normally distributed.

### Relevant MRI features

MRI features with a significant correlation (*p* value of ≤0.05) between the reference standard and at least two of three observers, and with a (weighted) kappa/intraclass correlation coefficient value of ≥0.60 for at least two of three observer pairs, were identified and considered potentially relevant in diagnosing proctitis. In post hoc analysis, threshold for the kappa/intraclass correlation in considering a relevant feature was changed to ≥0.40, because this concerns an initial study aimed at identifying potential relevant features and therefore sufficient features should be identified to be used in a future validation study.

All statistical analyses were performed with IBM SPSS Statistics version 20.0 for Windows (SPSS, Chicago, IL, USA) and Vassarstats.com (Richard Lowry, Poughkeepsie, NY, USA).

## Results

### Patient and MRI characteristics

**Fig. 2:**
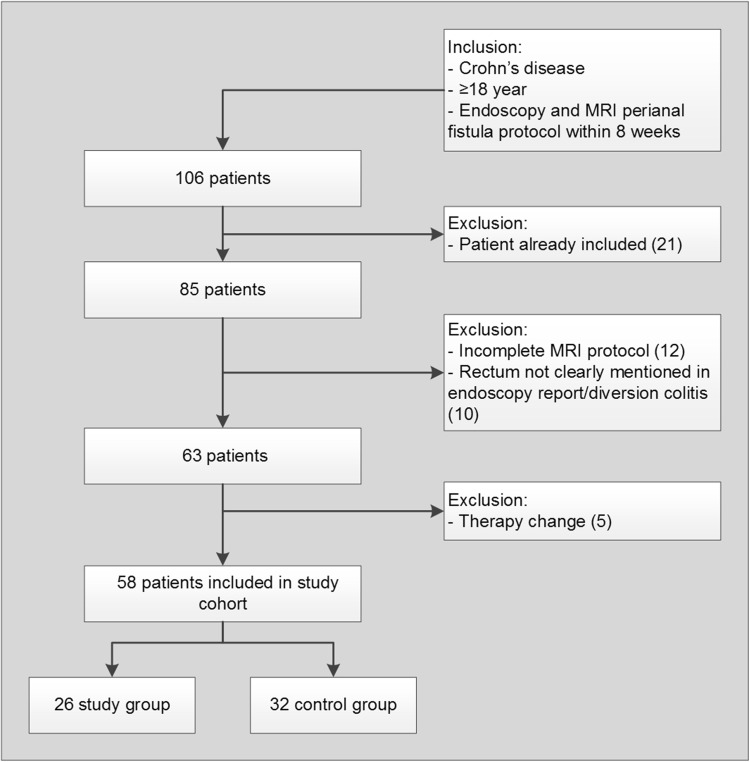
Flow chart of search in hospitals’ patient database

Between January 2001 and February 2014, 106 Crohn’s disease patients were extracted from the database who underwent perianal MRI within 8 weeks of endoscopy (Fig. 2). After exclusion, a total of 58 patients remained (Fig. 2), of which 21 (36%) are male with a mean age of 38.7 (SD 12.6) at the time of MRI. The median time between MRI and endoscopy was 12 days ([IQR 6–21]; range 0–44). Thirty-two (55%) had no signs of proctitis at endoscopy and 26 (45%) had signs of proctitis, of which 19 (33%) had non-ulcerative proctitis and 7 (12%) ulcerative proctitis. Table 2 summarized the demographic and clinical data of patients included in the study.

**Table 2:** Demographic characteristics of the study population

	Study group	Control group
No. (%) of patients	26 (55)	32 (45)
Men (%)	13 (50)	8 (25)
Women (%)	13 (50)	24 (75)
Age at time of imaging (y), median (IQR)	40 (27–51)	37 (28–47)
Disease duration (y), median (IQR)	7 (4–11)	7 (3–21)
Days between endoscopy and MRI, median (IQR)	13 (7–23)	11 (4–20)
Previous surgery, no. (%) of patients	16 (62)	22 (69)
Maintenance therapy, no. (%) of patients	18 (69)	23 (72)
Antitumor necrosis factor, no. (%) of patients	5 (19)	11 (34)
Steroids, no. (%) of patients	8 (31)	3 (9)
Immunosuppressant, no. (%) of patients	12 (46)	12 (38)
5-Aminosalicylic acid medications, no. (%) of patients	1 (4)	7 (22)
Vedolizumab, no. (%) of patients	1 (4)	0
Presence of fistula (on MRI)		
None, no. (%) of patients	3 (12)	8 (25)
Simple, no. (%) of patients	11 (42)	16 (50)
Complex, no. (%) of patients	12 (46)	8 (25)
Endoscopy diagnosis		
Absence of lesions, no. (%) of patients	0	32 (100)
Non-ulcerative lesions, no. (%) of patients	7 (27)	0
Ulcerations, no. (%) of patients	19 (73)	0

*IQR* interquartile range

Quality of the MRI scans was considered good in 74.1% (43/58) and adequate in 25.9% (15/58). There was no rectal distention in 43.1% (25/58), moderate distention in 34.5% (20/58), and good distention in 22.4% (13/58).

### Interobserver agreement

Agreement between the three observer pairs is presented in Table 3. For size of mesorectal lymph nodes, the agreement for all three pairs ranged between good and very good (0.78 and 0.83). Wall thickness, mural fat, and creeping fat showed good agreement for two of three observer pairs (0.70–0.58–0.69, 0.67–0.57–0.64, and 0.48–0.69–0.76, respectively). Perimural T2 signal, supralevatoric extension of fistula, and abscess showed at least moderate agreement (≥0.40) for all the three observer pairs. Perimural enhancement and size of inguinal lymph nodes showed at least moderate agreement for two of three observer pairs (0.46–0.34–0.59 and 0.65–0.38–0.43).

**Table 3:** Multirater Kappa and intraclass correlation coefficient values between the observer pairs

MRI features	1 vs. 2	2 vs. 3	1 vs. 3
Wall thickness	0.70 (0.54–0.81)	0.58 (0.38–0.73)	0.69 (0.53–0.81)
Mesorectal lymph nodes	0.83 (0.72–0.89)	0.78 (0.66–0.87)	0.83 (0.72–0.89)
Obturator lymph nodes	0.31 (0.06–0.53)	0.26 (0.00–0.48)	0.27 (0.01–0.49)
Iliac lymph nodes	0.38 (0.14–0.58)	0.23 (−0.03–0.46)	0.24 (−0.01–0.47)
Inguinal lymph nodes	0.65 (0.48–0.78)	0.38 (0.14–0.58)	0.43 (0.20–0.62)
% of circumference involved	0.16 (0.03–0.29)	0.17 (0.06–0.27)	0.47 (0.27–0.67)
Mural T2 signal	26% (15/58)^a^	43% (25/58)^a^	41% (24/58)^a^
Perimural T2 signal	0.50 (0.34–0.67)	0.57 (0.41–0.73)	0.71 (0.58–0.85)
T1 enhancement	0.13 (0.03–0.24)	0.14 (0.01–0.27)	0.39 (0.22–0.56)
T1 enhancement pattern	0.13 (0.02–0.25)	0.25 (0.12–0.38)	0.43 (0.25–0.61)
Perimural enhancement	0.46 (0.29–0.64)	0.34 (0.17–0.50)	0.59 (0.42–0.76)
Mural fat	0.67 (0.44–0.90)	0.57 (0.34–0.81)	0.64 (0.37–0.90)
Ulcers	64% (37/58)^a^	0.13 (0–0.39)	0.25 (0–0.57)
Supralevatoric fistula	0.48 (0.24–0.72)	0.57 (0.31–0.82)	0.59 (0.39–0.79)
Supralevatoric abscess	0.53 (0.21–0.86)	0.53 (0.24–0.82)	1
Creeping fat	0.48 (0.18–0.77)	0.69 (0.46–0.92)	0.76 (0.53–0.98)
Comb sign	0.18 (0.01–0.34)	0.20 (0.03–0.36)	0.55 (0.32–0.78)

^a^Proportion of agreement calculated instead of kappa, because observed concordance is smaller than mean-chance concordance

### Comparison of observers with reference standard

In Table 4, the comparison of continuous variables (upper part) and ordinal variables (lower part) with the reference standard are presented. Wall thickness was significantly smaller for all three observers in patients without proctitis than in patients with proctitis (observer 1: 6.0 vs. 9.0 mm, *p* = 0.000; observer 2: 8.0 vs. 11.0 mm, *p* = 0.023; observer 3: 4.0 vs. 10.0 mm, *p* = 0.000).

**Table 4:** Comparison of observers with reference standard

MRI Features		Observer 1		Observer 2		Observer 3	
		Median (IQR)	*p* value	Median (IQR)	*p* value	Median (IQR)	*p* value
Wall thickness (mm)	Normal	6.0 [4.0–8.0]	0.000	8.0 [7.0–12.0]	0.023	4.0 [3.0–6.8]	0.000
	Proctitis	9.0 [7.0–10.5]		11.0 [9.8–12.0]		10.0 [7.0–12.3]	
Mesorectal lymph nodes (mm)	Normal	3.0 [2.0–4.8]	0.001	4.0 [0.0–5.8]	0.005	3.0 [0.5–5.0]	0.000
	Proctitis	5.0 [4.0–7.0]		6.0 [4.8–7.3]		6.0 [4.0–8.0]	
Obturator lymph nodes (mm)	Normal	5.0 [4.0–7.0]	0.647	5.0 [0.0–6.0]	0.535	4.0 [3.0–5.0]	0.905
	Proctitis	6.0 [2.3–7.0]		5.0 [2.3–6.0]		4.0 [3.0–5.3]	
Iliac lymph nodes (mm)	Normal	5.0 [0.0–7.0]	0.375	5.0 [4.0–7.0]	0.855	0.0 [0.0–4.0]	0.403
	Proctitis	6.0 [4.0–7.0]		6.0 [0.0–7.0]		0.0 [0.0–4.3]	
Inguinal lymph nodes (mm)	Normal	8.0 [7.0–9.8]	0.442	8.0 [7.0–9.8]	0.427	8.0 [6.0–10.0]	0.575
	Proctitis	7.0 [6.0–10.0]		7.0 [6.0–9.0]		7.0 [6.0–10.0]	

MRI Features	Observer 1		Observer 2		Observer 3	
	Exp(B) (95% CI)	*p* value	Exp(B) (95% CI)	*p* value	Exp(B) (95% CI)	*p* value
% of circumference involved	14.18 (3.60–55.87)	0.000	12.65 (2.60–61.65)	0.002	9.90 (2.96–31.09)	0.000
Mural T2 signal	4.34 (1.58–11.91)	0.004	3.16 (1.07–9.37)	0.038	3.90 (1.07–16.30)	0.062
Perimural T2 signal	20.43 (4.13–100.99)	0.000	4.79 (1.72–13.34)	0.003	20.25 (4.95–85.76)	0.000
T1 enhancement	3.10 (1.15–8.18)	0.025	2.16 (1.30–6.10)	0.145	2.69 (1.01–7.17)	0.049
T1 enhancement pattern	2.48 (1.05–6.46)	0.064	6.28 (2.00–19.71)	0.002	2.89 (1.11–7.57)	0.030
Perimural enhancement	11.70 (3.27–41.85)	0.000	9.06 (2.81–29.22)	0.000	17.76 (4.35–74.07)	0.000
Mural fat	6.67 (1.28–34.92)	0.025	2.23 (0.71–7.06)	0.172	16.41 (1.91–140.75)	0.011
Ulcers	0.47 (0.11–2.02)	0.307	1.11 (0.34–3.60)	0.868	9.30 (1.04–83.13)	0.046
Supralevatoric fistula	1.25 (0.44–3.56)	0.673	1.60 (0.46–5.52)	0.460	1.93 (0.57–6.51)	0.291
Supralevatoric abscess	5.64 (0.59–53.93)	0.133	3.11 (0.82–11.86)	0.096	5.64 (0.60–53.93)	0.133
Creeping fat	^a^		7.09 (1.71–29.35)	0.007	16.41 (1.91–140.75)	0.011
Comb sign	7.00 (1.91–25.67)	0.003	5.96 (1.48–23.97)	0.012	13.18 (3.18–54.57)	0.000

^a^Not calculated because of the presence of a zero in the crosstab

Mesorectal lymph nodes were smaller for all three observers in patients without proctitis than with proctitis (observer 1: 3.0 vs. 5.0 mm, *p* = 0.001; observer 2: 4.0 vs. 6.0 mm, *p* = 0.005; observer 3: 3.0 vs. 6.0 mm, *p* = 0.000).

Percentage of circumference involved, perimural T2 signal, perimural enhancement, and the presence of the comb sign showed also a significant correlation between all three observers and the endoscopy reference standard (Table 4).

Mural T2 signal, T1 enhancement, T1 enhancement pattern, and creeping fat showed a significant correlation for two of three observers. Ulcers and supralevatoric extension of fistula and abscess did not show a significant correlation to the reference standard for all three observers.

### Relevant MRI features

**Fig. 3:**
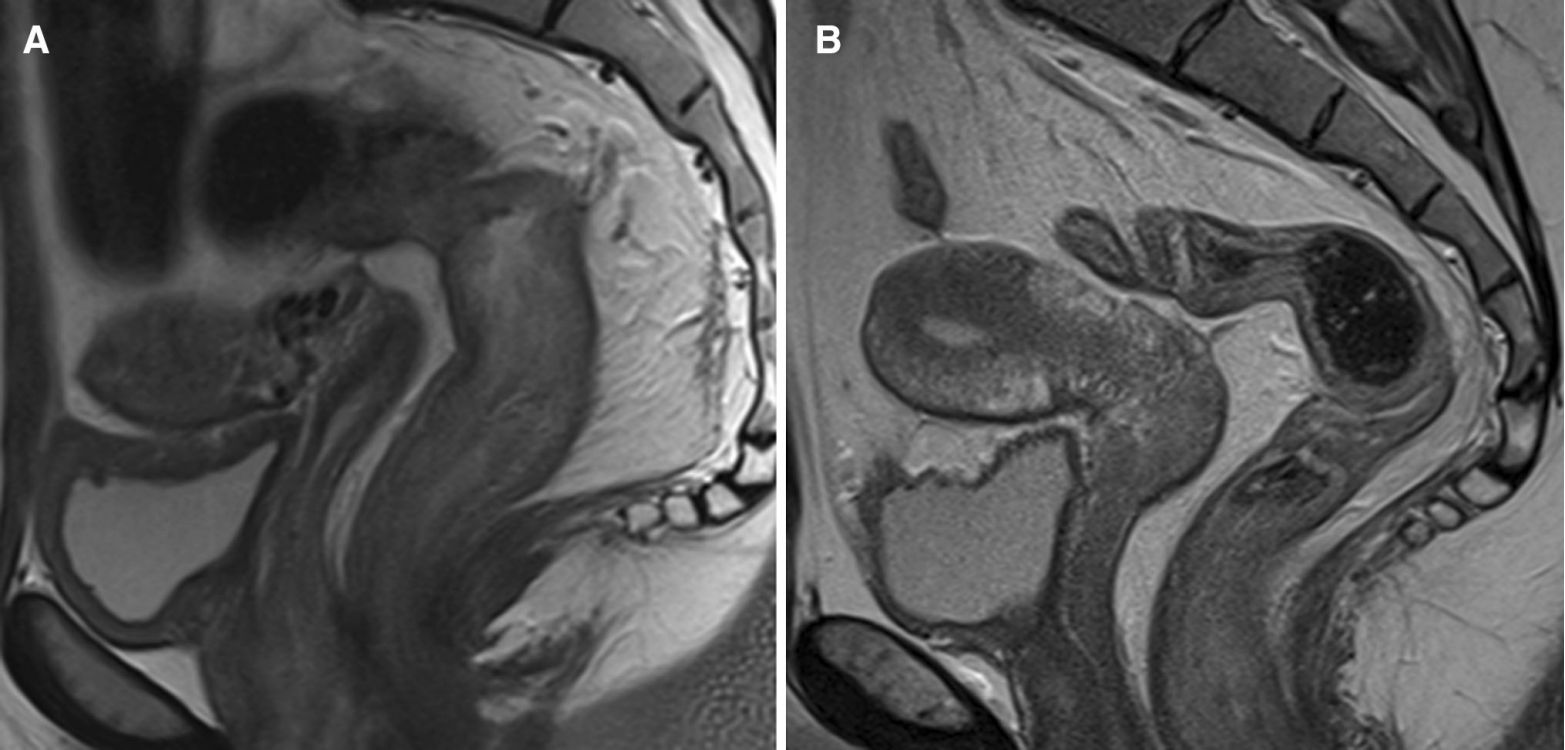
Sagittal T2-weighted image of two different patients with Crohn’s disease. **A** A 25-year-old female with ulcerative proctitis at endoscopy. The image shows increased amount of mesorectal fat tissue (creeping fat) and a subtle increase of perimural vascularity (‘comb sign’) in addition to rectal wall thickening. **B** A 24-year-old female with no signs of proctitis at endoscopy. There is no increased amount of mesorectal fat tissue and the rectum shows no abnormal MRI features

**Fig. 4:**
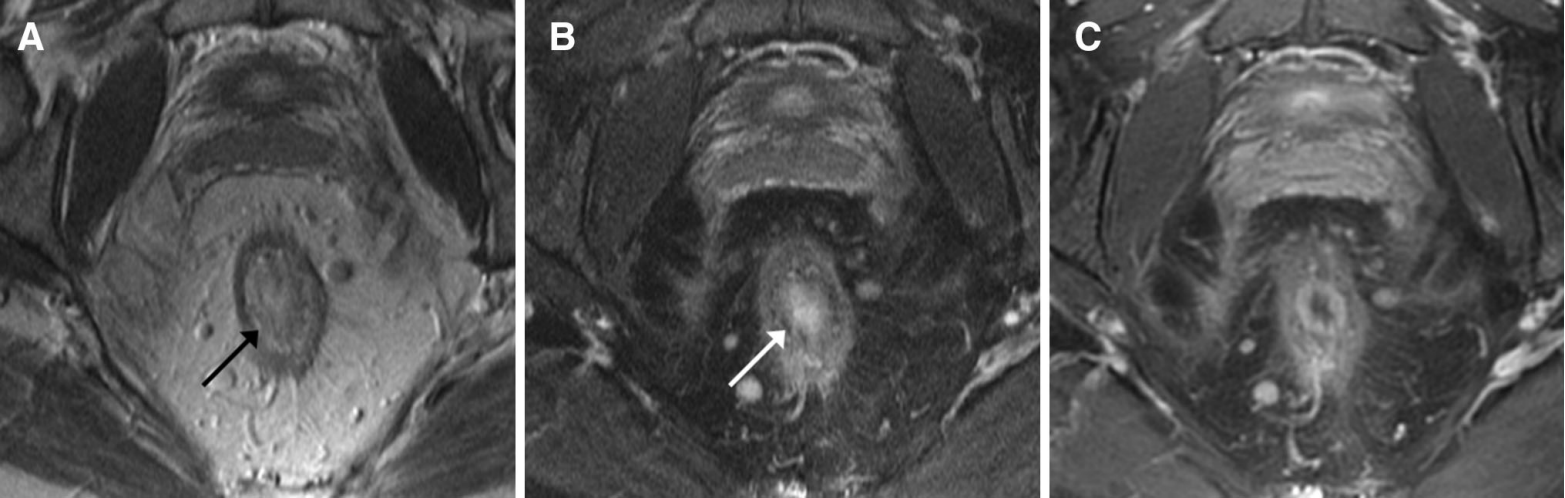
A 53-year-old female with Crohn’s disease and ulcerative proctitis at endoscopy. **A** Axial oblique T2-weighted image shows high mural signal intensity and **B** low signal intensity on axial oblique fat-saturated T2-weighted image corresponding to mural fat (*arrow*). **C** Axial oblique fat-saturated post-contrast T1-weighted images shows moderate enhancement of the rectal wall and perimural fat tissue. In addition, wall thickening and multiple mesorectal lymph nodes are present

**Fig. 5:**
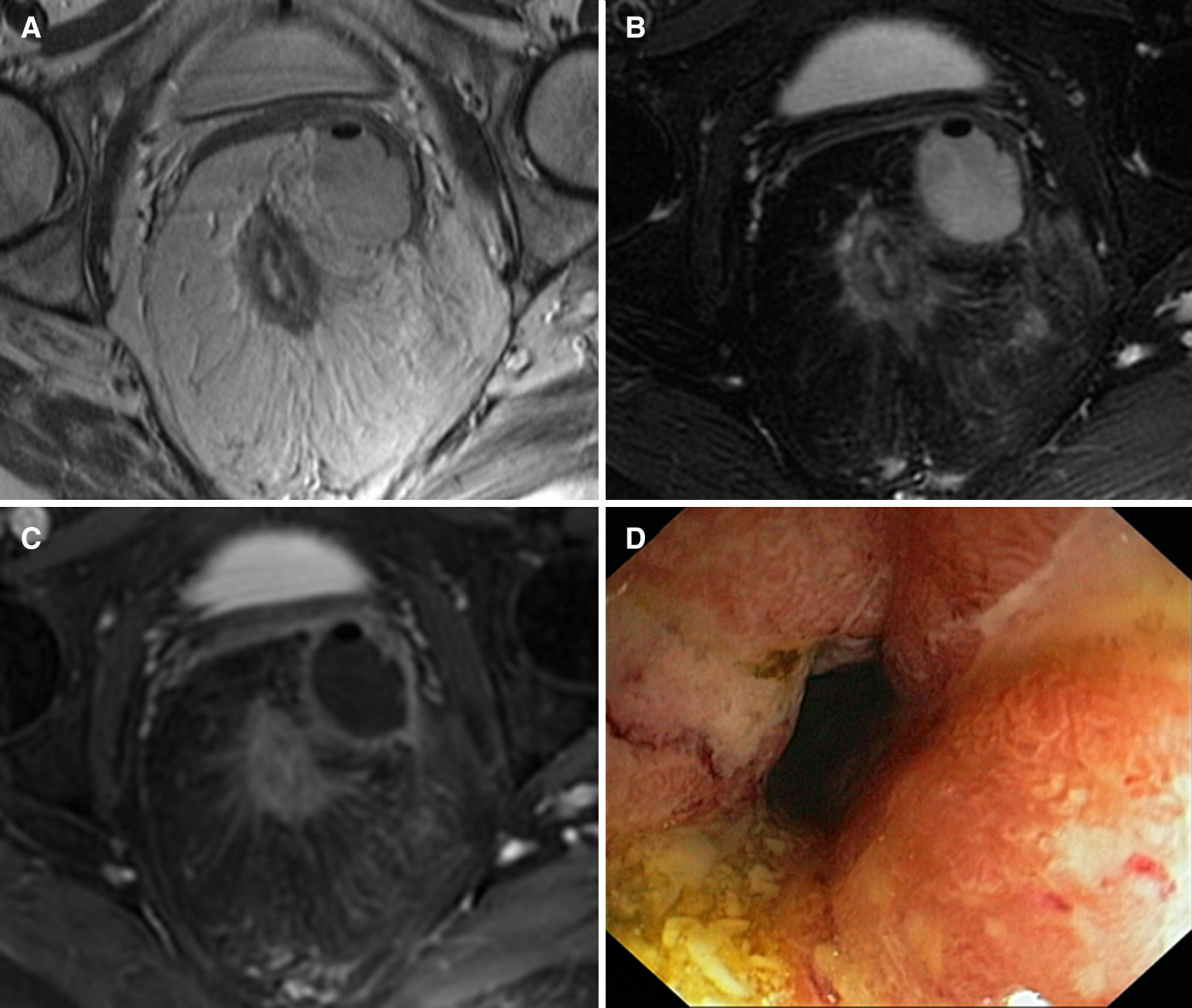
A 49-year-old female with Crohn’s disease. **A** Axial oblique T2-weighted image and **B** axial oblique fat-saturated T2-weighted image show rectal wall thickening, a marked increase of T2 signal intensity and a perimural large fluid rim (>2 mm). **C** Axial oblique fat-saturated post-contrast T1-weighted images obtained at the same level shows a moderate enhancement of the rectal wall and the perimural fat tissue. In addition, there is creeping fat and a supralevatoric abscess left anterolateral of the rectum on all three images. **D** Endoscopy showed ulcerative proctitis

Based on predefined criteria, the following MRI features were considered most relevant in diagnosing proctitis (Tables 3, 4): wall thickness, size of mesorectal lymph nodes, mural fat, and creeping fat showed a significant correlation between at least two of three observers and the endoscopy reference standard, as well as a kappa/intraclass correlation coefficient of ≥0.60 for at least two of three observer pairs (Figs. 3, 4). In post hoc analysis, a kappa/intraclass coefficient threshold of ≥0.40 was considered, which included perimural T2 signal and perimural enhancement as they showed a moderate interobserver agreement for two of three observer pairs and a significant correlation with the reference standard for all three observers (Appendix B) (Fig. 5).

All other features (all other lymph nodes, % of circumference involved, T1 enhancement (pattern), ulcers, supralevatoric fistula, and abscess and comb sign) did not fulfill our predefined criteria for relevancy. Although the correlation with endoscopy was significant for two observers, mural T2 signal and T1 enhancement (pattern) showed poor to moderate agreement.

## Discussion

MRI features rectal wall thickness, mesorectal lymph nodes, mural fat, and creeping fat were considered relevant in diagnosing proctitis on pelvic MRI, as they showed a significant correlation between at least two observers and the endoscopy reference standard, and at least a good interobserver agreement for at least two of three observer pairs. Perimural T2 signal and perimural enhancement showed a significant correlation for all the three observers and a moderate interobserver agreement for at least two of the three observer pairs. Mural T2 signal and T1 enhancement degree and pattern showed poor to moderate reproducibility.

This is to our knowledge, the first study reporting on the specific MRI features associated with proctitis on a dedicated pelvic MRI. Previous research did study rectal involvement in Crohn’s disease patients, but this was done using MR enterography or MR colonography [8–10]. No specific rectal and/or perirectal features were described. Van Assche developed an MRI-based score of perianal Crohn’s disease severity with rectal wall thickening as the sole indicator for rectal inflammation, which can be used for evaluation of response to treatment [16, 17]. Our results confirmed the correlation between rectal wall thickening and inflammation; in addition, a moderate to good interobserver agreement was observed. Most features considered relevant in diagnosing proctitis involved the mesorectal fat tissue (Figs. 4, 5). This is in contrast to a study that showed only fair reproducibility for perimural features on MR enterography [11]. Crohn’s disease is known for its transmural inflammation and subsequent perimural involvement. In the rectum, this perimural involvement was often quite prominent. This might be related to the isolated localization of the rectum surrounded by mesorectal fat tissue, where perimural changes are somewhat easier appreciated than when multiple loops of bowel are closely aligned. Further, the rectum was not or moderately distended in most cases, which might result in the perirectal features becoming more apparent. This rectal collapse might have led to increased wall thickness measurements. Even so, there was a significant difference in wall thickness in proctitis versus no proctitis as observed by all three observers. Further research should focus on the predictive value of the individual MRI features identified in our study, and the clinical use in monitoring treatment response as a non-invasive alternative to endoscopy and in case of severe anal stenosis.

**Fig. 6:**
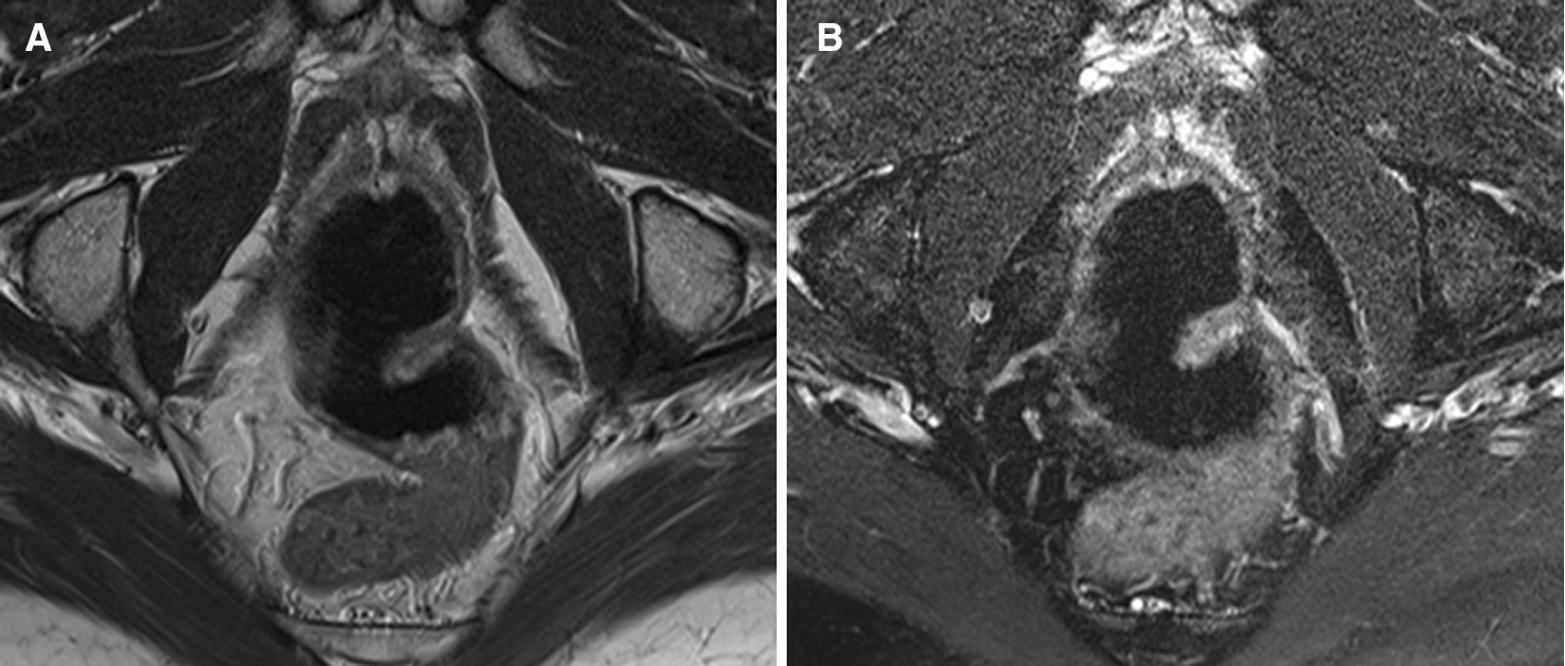
A 45-year-old male with Crohn’s disease and no signs of proctitis at endoscopy. **A** Axial oblique T2-weighted image and **B** axial oblique fat-saturated T2-weighted image obtained at the same level shows a moderate increase of T2 signal intensity of the rectal wall

In contrast, luminal features already proven to be useful in establishing disease severity on MR enterography and MR colonography, for example, T2 signal intensity and T1 enhancement (pattern and degree), were considered not useful in our study [7–9]. In order to decide if a certain feature is considered normal or increased, one must be able to compare it to other colonic loops, which were almost never included in the field of view of the T1-weighted and T2-weighted fat-saturated sequences that was only performed in the axial oblique plane. Also, in our standard perianal fistula protocol, no T1-weighted pre-contrast images for comparison were performed. For the T2 signal intensity of the rectal wall in almost all cases (normal or proctitis), observers scored the T2 signal intensity of the rectal wall at least as slight, but mostly as moderately increased (Fig. 6). This suggests that the normal rectal signal intensity is already light gray on T2 fat-saturated images and that the grading scale used for luminal disease was not adequate for the rectum. Furthermore, MR enterography and colonography use luminal contrast to obtain bowel distention.

**Fig. 7:**
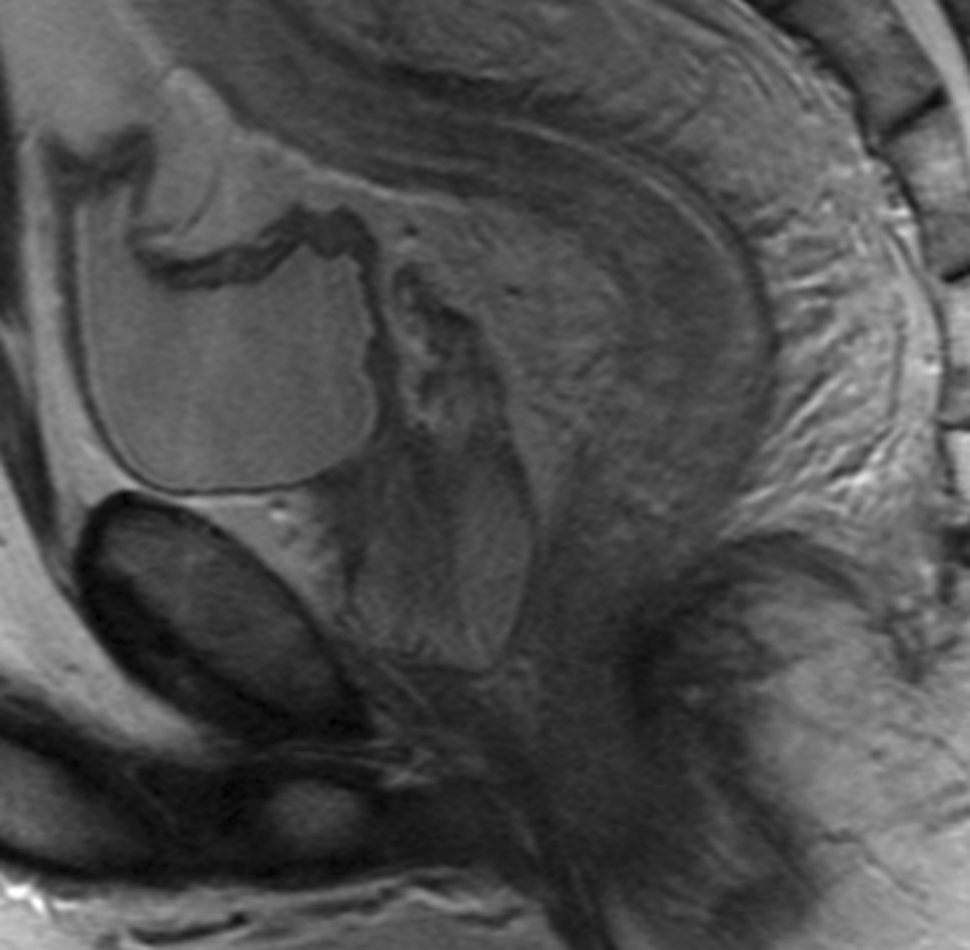
A 43-year-old male with Crohn’s disease and ulcerative proctitis at endoscopy. Sagittal T2-weighted image shows the increased perimural vascularity perpendicular to the rectum (‘comb sign’) in addition to the wall thickening of the entire rectum

We do not have a balanced explanation for the lack of correlation between the supralevatoric extension of fistula and/or abscess and the presence of proctitis as one might have expected. Only our most experienced observer showed a significant correlation for the presence of ulcerations. A limitation for this feature is that in the proctitis group, we combined the patients with non-ulcerative and ulcerative proctitis because of low number of patients in each group. As the number of ulcerative proctitis cases was low, a possible correlation in ulcerative proctitis might not be identified. Combining ulcerative and non-ulcerative proctitis, also prevented us to rule on disease severity. For the features ‘presence of creeping fat’ and ‘comb sign,’ we had no circumscribed definitions or grading, and observers had to score these features according to their expert opinion rendering it susceptible for subjectivity. Although the comb sign did show a significant correlation with endoscopy, the interobserver agreement was very low for two of three observer pairs, probably because of unfamiliarity with this feature (Fig. 7).

Increased wall thickness is not only seen in the active phase of Crohn’s disease but also in the chronic stage of the disease. Also, the presence of mural fat and creeping fat are signs of chronic disease. The significant correlation to active inflammation at endoscopy for these features is inherent to Crohn’s disease with a chronic course of relapsing and remitting inflammation, where features of chronic disease coexist with acute inflammatory changes.

Our study has several limitations. First, endoscopy reports were retrospectively analyzed in order to determine the reference standard. However, only reports with evident mentioning of the rectum were included and evaluated using a predefined clear-cut scoring system [10] and an experienced gastroenterologist was involved. Because of only including patients with mentioning of the rectum in the endoscopy report, our patient population was subject to selection bias creating a disease-enriched population. Second, the time between the MRI and the endoscopy examination ranged from 0 to 44 days. In this time frame disease activity could have been altered because of natural course. However, since we excluded all patients with change in medical or surgical therapy during the time interval, this possibility was minimized. Third, the use of a kappa/intraclass coefficient value of ≥0.60 would have strengthened our study, but at initial evaluation of our data too little features remained. In this first phase of identifying possible relevant features, we wanted to include as much features as possible, in order to make a further selection in a future validation study. Fourth, the introductory session for the observers regarding the different MRI features was held by the same expert abdominal radiologist (JS who was also one of the readers in this study), which could have increased reproducibility. We did not notice higher agreement between the observer pairs including this expert abdominal radiologist than the other observer pair.

In conclusion, for diagnosing proctitis in Crohn’s disease in perianal MRI, MRI features involving the mesorectal tissue, perimural T2 signal, perimural T1 enhancement, the presence of creeping fat, and size of mesorectal lymph nodes were most valuable, as they showed a significant correlation with endoscopical findings and were reproducible. Established luminal features like mural T2 signal and T1 enhancement may be less helpful in perianal MRI. In addition, rectal wall thickness and presence of mural fat were relevant as well. Future research should focus on these MRI features by validating them in a prospective study and for defining thresholds for continuous variables.

## Appendices

**Appendix A:** Range of MR scan parameters performed on three different MR scanners

	Sagittal T2-weighted TSE	Coronal T2-weighted TSE	Axial T2-weighted TSE	Axial T2-weighted TSE with fat sat.	Axial T1-weighted TSE with fat sat. + iv contrast enhancement^a^
1.5 T (Siemens, Avanto), 33 MR scans					
Field of view (cm)	300	300/320	220/300	220/300	300–450
No. of slices	28–40	25–42	28–45	28–42	28–42
Repetition time (ms)	2500–4000	2500–4000	2500–4000	2500–4000	600–718
Echo time (ms)	69/70	70–121	70–121	70–121	9,4/11
Image matrix	512 × 231−512 × 578	256 × 297−512 × 575	256 × 297−512 × 578	256 × 297−512 × 575	256 × 288−512 × 256
Slice thickness (mm)	3/4	3/4	3/4	4	4
Slice gap (mm)	0.4	0–0,4	0–0.4	0/0.4	0–0,4
NSA	1–3	1/2	1/2	1/2	1–4
Flip angle	90/150	90/150	90/150	90/150	90/150
1.5 T (Signa, GE), 20 MR scans					
Field of view (cm)	300	300	300	300	300/450
No. of slices	32	32	31/32	28–32	31/32
Repetition time (ms)	2500	2500	2500	2500–4500	560/600
Echo time (ms)	69/70	71/72	69	69–83	10.8
Image matrix	512 × 200	512 × 200	512 × 200	256 × 100	256 × 100
Slice thickness (mm)	4	4	4	4	4
Slice gap (mm)	0.4	0.4	0.4	0.4	0.4
NSA	1	1	1	1	2
Flip angle	90	90	90	90	90
3 T (Philips), 5 MR scans					
Field of view (cm)	230–360	240–400	240–400	240–400	300/400
No. of slices	30–41	30–37	32–45	35–45	40–45
Repetition time (ms)	2689/3000	2689–3000	2689–3000	3000/4626	550–786
Echo time (ms)	70–100	70–100	70–100	70/100	10
Image matrix	256 × 198−528 × 361	400 × 246−528 × 400	400 × 395−528 × 361	300 × 287−768 × 287	248 × 248−512 × 255
Slice thickness (mm)	3/4	3/4	3/4	3/4	3/4
Slice gap (mm)	0–1	0/0.3	0/0.3	0/0.3	0/0.3
NSA	2/4	1/4	1/4	1/2	1/2
Flip angle	90	90	30	90	90

^a^With the exception of one SENSE dixon post-contrast series: FOV 400, no. of slices 480, TR 0, TE 0, matrix 512x207, slice thickness 1.5, gap 0, averages 1, flip angle 8

**Appendix B:** Combined results of interobserver agreement and correlation between observers and reference standard

MRI features	Significant correlation between observer and reference standard (*p* ≤ 0.05)			Kappa or intraclass coefficient ≥0.40			Relevant MRI feature
	Observer 1	Observer 2	Observer 3	1 vs. 2	2 vs. 3	1 vs. 3	
Wall thickness	Yes	Yes	Yes	Yes	Yes	Yes	Yes
Mesorectal lymph nodes	Yes	Yes	Yes	Yes	Yes	Yes	Yes
Obturator lymph nodes	No	No	No	No	No	No	No
Iliac lymph nodes	No	No	No	No	No	No	No
Inguinal lymph nodes	No	No	No	Yes	No	Yes	No
% of circumference involved	Yes	Yes	Yes	No	No	Yes	No
Mural T2 signal	Yes	Yes	No	No	No^a^	No^a^	No
Perimural T2 signal	Yes	Yes	Yes	Yes	Yes	Yes	Yes
T1 enhancement	Yes	No	Yes	No	No	No	No
T1 enhancement pattern	No	Yes	Yes	No	No	Yes	No
Perimural enhancement	Yes	Yes	Yes	Yes	No	Yes	Yes
Mural fat	Yes	No	Yes	Yes	Yes	Yes	Yes
Ulcers	No	No	Yes	No^a^	No	No	No
Supralevatoric fistula	No	No	No	Yes	Yes	Yes	No
Supralevatoric abscess	No	No	No	Yes	Yes	Yes	No
Creeping fat	^b^	Yes	Yes	Yes	Yes	Yes	Yes
Comb sign	Yes	Yes	Yes	No	No	Yes	No

^a^Proportion of agreement calculated, because observed concordance is smaller than mean-chance concordance. Assuming kappa ≤0.40

^b^Not calculated because of the presence of a zero in the crosstab
